# Lysosomal mTORC2/PHLPP1/Akt axis: a new point of control of chaperone-mediated autophagy

**DOI:** 10.18632/oncotarget.5903

**Published:** 2015-09-28

**Authors:** Esperanza Arias

**Affiliations:** Department of Developmental and Molecular Biology, Albert Einstein College of Medicine, Bronx, NY, USA

**Keywords:** lysosomes, membrane proteins, kinase, phosphatase, cell signaling

Chaperone-mediated autophagy (CMA), a mechanism for degradation of cytosolic proteins in lysosomes, is one of the most selective types of autophagy in mammals [1]. Identification and degradation of single proteins via CMA allows precise remodeling of the cellular proteome and removal of altered proteins. These characteristics explain the contribution of CMA to the cellular response to stress, in cellular quality control and in the fine-tune regulation of processes such as cellular metabolism, differentiation and reprograming [1]. The molecular components that participate in substrate targeting and translocation across the lysosomal membrane in CMA are well characterized [1]. In contrast, the signaling mechanisms that contribute to its regulation were poorly understood. Until recently only signaling through the retinoic acid receptor α had been identified to negatively regulate CMA activity [2]. In T cells, nuclear factor of activated T-cells (NFAT) signaling mediates the upregulation of CMA required for T cell activation, but it remains unknown whether or not this regulation is T cell specific [3].

Despite the unique functions attributed to CMA, this pathway does not act in isolation. Quite on the contrary, growing evidence supports coordinated functioning of the different autophagic pathways that co-exist in the cell and direct cross-talk between macroautophagy and CMA (both maximally activated in response to stress). Thus, CMA-defective cells maintain protein degradation at normal levels through macroautophagy up-regulation [1]. Similarly, blockage of macroautophagy results in activation of CMA even under basal conditions [1]. Although these pathways are not redundant and the deficit of the blocked pathway becomes evident upon stress, this partial compensation serves to maintain cellular homeostasis under basal conditions. Consequently, understanding the basis of this bi-directional cross-talk between macroautophagy and CMA becomes important, especially in pathological conditions with blockage of one of these autophagic pathways.

In our search for signaling mechanisms that regulate CMA and that could also participate in the autophagic crosstalk, we focused our attention on the mammalian target of rapamycin (mTOR), a serine/threonine kinase that serves as one of the main cellular nutritional sensors. mTOR senses and integrates different nutritional inputs, including growth factors, energy levels, cellular stress, and amino acids. mTOR is present in two distinct complexes, mTORC1 and mTORC2. There is extensive evidence of a tight relation of mTORC1 with lysosomes and macroautophagy regulation. Amino acids signal to mTORC1, and allow its translocation to the lysosomal surface where it becomes activated. This process is mediated by the coordinated actions of multiple complexes, including the Ragulator, and Rag GTPases [4]. Activation of lysosomal mTORC1 results in macroautophagy inhibition.

**Figure 1:**
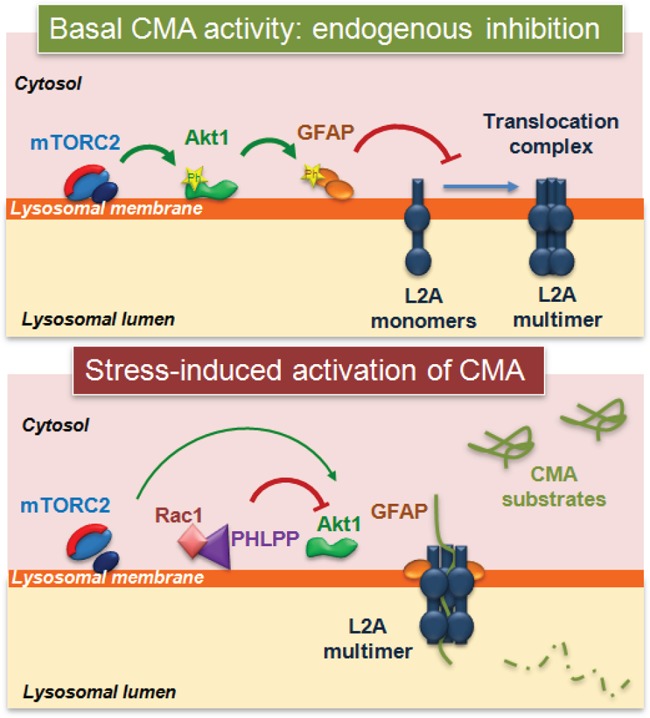
Chaperone-mediated autophagy activity is regulated by the phosphorylation state of lysosomal Akt Phosphorylation of lysosome-associated Akt by mTORC2 represses CMA activation by negatively regulating the assembly of LAMP-2A into the CMA translocation complex, in part through phosphorylation of GFAP. Activation of CMA in response to stress is attained by the recruitment to lysosomes of the phosphatase PHLPP1 (that dephosphorylates Akt) and the GTPase Rac1 (that stabilizes PHLPP1 at the membrane). Reduced Akt activity enhances CMA by increasing the stability of the CMA translocation complex at the lysosomal membrane.

Interestingly, we have recently identified that mTOR is also involved in CMA regulation [5] Figure 1. CMA is under the negative regulation of mTORC2 and its kinase substrate Akt1. Both regulatory kinases bind and act directly at the membrane of the subset of lysosomes involved in CMA. Continue signaling through mTORC2 under basal conditions is behind the relatively low levels of constitutive CMA activity in most cells. Release of the inhibitory effect of mTORC2/Akt1 and subsequent activation of CMA is attained through the association with the lysosomal membrane of the phosphatase PHLPP1 that inactivates Akt1 by dephosphorylation. The GTPase Rac1 also associates with the lysosomal membrane under these conditions and contributes to stabilize PHLPP in this compartment. Lysosomal recruitment of PHLPP to neutralize mTORC2 activity could appear unnecessary during nutrient starvation, since mTORC2 activity is reduced under these conditions. However, we found that mTORC2 activity is only inhibited early during starvation, but the kinase becomes fully active as starvation persists. Consequently, PHLPP is needed at the lysosomal membrane to oppose the mTORC2 inhibitory effect on CMA, which reaches maximal activation at 15-20h of starvation. The modulatory effect of the mTORC2/PHLPP1/Akt axis on CMA is exerted by modifying the kinetics of assembly/disassembly of the CMA translocation complex in the lysosomal membrane and it takes place entirely in the lysosomal compartment, as it can be fully reproduced in isolated lysosomes *in vitro*.

The physiological relevance of this novel mechanism for CMA regulation is easily inferred in the context of the recently discovered role of CMA in metabolic control. Mice defective for CMA in liver [6] display marked deficiencies in glucose and lipid metabolism that manifest as hepatic glycogen depletion and hepatosteatosis. These metabolic alterations are due to the fact that CMA degrades in timely manner enzymes involved in carbohydrate and lipid metabolism, thus contributing to modulate flux through these metabolic pathways [6]. In our latest study, we demonstrate that pharmacological inhibition of mTORC2 in the presence of nutrients is sufficient to downregulate glycolysis to levels similar to those observed during starvation and that this effect is fully dependent on the activation of CMA. These results highlight the potential value of modulating CMA activity as a novel way to control glucose metabolism in pathological conditions [5].

The study of the mTORC2/PHLPP1/Akt pathway in the physiological regulation of CMA has also shed light on the unique nature of CMA regulation in cancer cells. Several studies have demonstrated constitutive high levels of CMA activity in many cancer cell types and that this CMA upregulation is required for maintenance of the metabolic alterations of malignant cells such as the Warburg effect [7]. This constitutive activation of CMA in cancer contrasts with the common upregulation of *AKT* and frequent loss of the *PHLPP1* loci in many cancers types. We found that contrary to normal cells, CMA in cancer cells was insensitive to blockage of mTORC2 or Akt1 and could not be repressed by inhibition of PHLPP1. These findings support that cancer cells may have developed unique mechanisms to desensitize CMA from the inhibitory effect of mTORC2/Akt1 and, in this way, support sustained activation of this autophagic pathway. Understanding the basis of this desensitization may help identifying novel antioncogenic therapeutic targets.

Overall, our study reveals the lysosomal mTORC2/PHLPP1/Akt axis as a point of control of CMA that could be modulated to restore CMA dysfunction in disease, including cancer and metabolic disorders.
